# How virus migration and meteorological elements shape the seasonality of influenza a/H3N2: A case study in China

**DOI:** 10.1016/j.onehlt.2025.101037

**Published:** 2025-04-14

**Authors:** Yilan Liao, Tong Zhao, Wei Du, Dayan Wang, Zhibin Peng, Shan Xue, Jianxing Yu, An Zhang, Hongyan Ren, Zhoupeng Ren, Geoge Fu Gao, Jinfeng Wang, Nils Christian Stenseth

**Affiliations:** a State Key Laboratory of Resources & Environmental Information System, Institute of Geographic Sciences & Natural Resources Research, Chinese Academy of Sciences, Beijing 100101, China; b Jiangsu Center for Collaborative Innovation in Geographical Information Resource Development and Application, Nanjing 210023, China; c University of Chinese Academy of Science, Beijing 100049, China; d School of Public Health, Southeast University, Nanjing 210009, China; e Chinese National Influenza Center, National Institute for Viral Disease Control and Prevention, Chinese Center for Disease Control and Prevention, No. 155# Changbai Road, Changping District, Beijing 102206, China; f Division of Infectious Diseases, Chinese Center for Disease Control and Prevention, No. 155# Changbai Road, Changping District, Beijing 102206, PR China; g National Institute for Communicable Disease Control and Prevention, Chinese Center for Disease Control and Prevention, No.155# Changbai Road, Changping District, Beijing 102206, PR China; h CAS Key Laboratory of Pathogenic Microbiology and Immunology, Institute of Microbiology, Chinese Academy of Sciences, Beijing 100101, China; i CAS Key Laboratory of Pathogenic Microbiology and Immunology, Institute of Microbiology, Center for Influenza Research and Early-warning (CASCIRE), CAS-TWAS Center of Excellence, Emerging Infectious Diseases (CEEID), Chinese Academy of Sciences, Beijing 100101, China; j Savaid Medical School, University of Chinese Academy of Sciences, Beijing 100049, China; k Centre for Ecological and Evolutionary Synthesis (CEES), Department of Biosciences, University of Oslo, Oslo 0316, Norway; l One Health Center for Pandemic Research, Sustainable Health Unit (SUSTAINIT), Faculty of Medicine, University of Oslo, Oslo 0316, Norway

**Keywords:** Influenza seasonality, Virus migration, Meteorological elements, A/H3N2

## Abstract

Recent global influenza resurgences, escalating to pandemics, emphasize the urgency for effective vaccinations. Despite their efficacy, vaccines offer limited protection against A/H3N2 variants. Thus, elucidating the spatial patterns and underlying drivers of A/H3N2 seasonality is critical for its management. However, the mechanisms governing this seasonality are not fully understood. The study conducted a collaborative and interdisciplinary analysis of influenza A/H3N2 epidemiology in China from 2012 to 2018, utilizing national influenza surveillance data, viral gene sequence data, and meteorological information. We initially examined the spatiotemporal distribution of influenza A/H3N2 across different temperate zones in China. Subsequently, we employed Bayesian “SkyGrid” reconstruction analysis to gain insights into the population dynamics of the influenza A/H3N2 virus within China's temperature zones. Additionally, we utilized generalized additive models (GAM) to assess the influence of meteorological factors on the seasonal prevalence of influenza A/H3N2. Our analysis of China's national influenza data revealed distinct seasonal patterns for A/H3N2: winter epidemics prevailed in temperate zones, while summer and autumn outbreaks occurred in subtropical and tropical areas. The seasonality of influenza A/H3N2 across China's diverse climatic zones is shaped by the interplay of virus migration and meteorological factors. Virus migration introduced new variant populations during seasonal epidemics of influenza A/H3N2 to different temperature zones in China, thereby seeding subsequent seasonal outbreaks. Our findings also indicate that meteorological elements trigger influenza A/H3N2 activity following virus migration. Moreover, the spatial variations in influenza A/H3N2 seasonality in China can be attributed to specific temperature thresholds, approximately 1 °C and 24 °C. These thresholds could serve as potential indicators for A/H3N2 prevalence. This insight is invaluable for tailoring region-specific prevention and control strategies in China and other regions with similar environmental conditions.

## Highlights

• Understanding the spatial patterns and drivers of influenza A/H3N2 seasonality is crucial for addressing its public health challenges.
• Our study first examined virus migration and meteorological factors' combined impact on influenza A/H3N2 seasonality in China.
• Virus migration seeds influenza A/H3N2 variants, driving epidemics; weather shifts at 1°C and 24°C explain spatial seasonality in China.

## Graphical abstract

**Figure:**
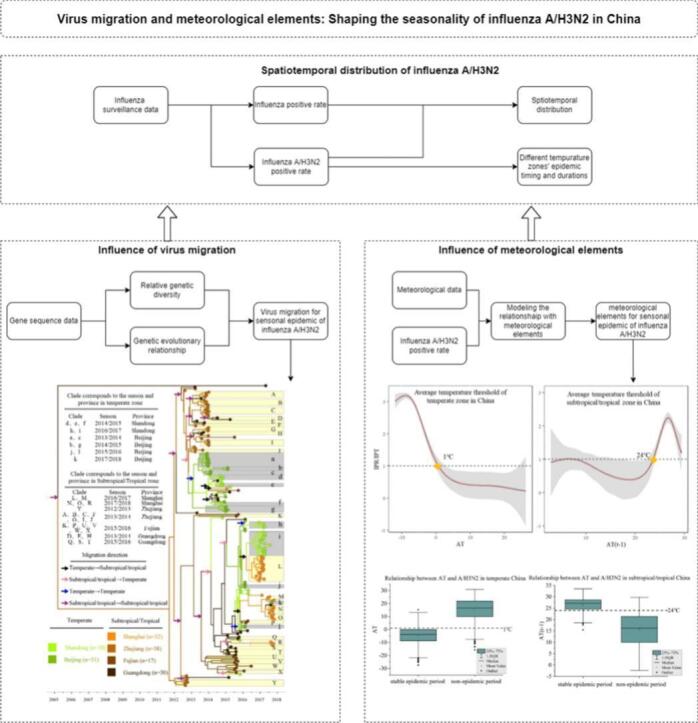

## 1 Introduction

Influenza is a widespread respiratory virus, infecting approximately one-fifth of the global population annually [1]. While seasonal influenza activity declined in 2020–21 due to the emergence of COV ID-19, it rebounded in 2021–22, exacerbating the challenges of influenza prevention and control [2]. Vaccination is widely recognized as the most effective measure against influenza infections [3]. However, studies have shown that influenza vaccines offer limited protection against A/H3N2-related illnesses compared to other influenza subtypes [4,5]. Therefore, effective prevention and control strategies for seasonal influenza A/H3N2 require a deeper understanding of its spatial distribution patterns and driving mechanisms.

In the temperate zone, influenza A/H3N2 follows a cyclical winter epidemic pattern [6,7], while the seasonality of influenza A/H3N2 in the subtropical/tropical zone remains less defined. According to data from the WHO's Global Influenza Surveillance and Response System (GISRS), influenza A/H3N2 is not just a winter epidemic and exhibits a more complex seasonality pattern in the subtropical/tropical zone compared to the temperate zone.

How human activities, animal populations, and environmental factors interact and contribute to the epidemic's dynamics? The driving mechanism behind influenza A/H3N2 seasonality remains elusive, with various hypotheses proposed in the literature. One hypothesis suggests that influenza A/H3N2 viruses originate in the subtropical/tropical zone, migrate to the temperate zone during epidemic periods, and return to the subtropical/tropical zone during non-epidemic periods [[8], [9], [10]]. However, challenges exist with this hypothesis, as it has been confirmed that the subtropical/tropical zone is not the sole origin of influenza A/H3N2 viruses, and the migratory pathways are irregular [11]. Alternatively, another hypothesis posits that environmental factors influence influenza A/H3N2 seasonality. Previous studies have identified correlations between influenza seasonality and meteorological factors, including temperature [12], humidity [13], precipitation [14], duration of sunshine [15], and wind speed [16]. These meteorological factors impact influenza activity by affecting virus transmission, survival, and host immunity and behavior [17]. However, the debate persists regarding whether the same climatic factors drive influenza A/H3N2 seasonality in different temperature zones, with varying results reported across different research regions. Key questions arise: What meteorological elements contribute to the spatial variability of influenza A/H3N2 seasonality worldwide? Is there a shared regulatory influence on virus migration and meteorological factors impacting influenza A/H3N2 seasonality? These critical questions warrant further investigation.

In our study, we operated under the assumption that influenza A/H3N2 viruses remain dormant and are activated by specific seasonal meteorological conditions [8]. China, encompassing both temperate and subtropical/tropical zones, offered an ideal setting for this research. Leveraging the Chinese National Influenza Surveillance Network, we analyzed weekly influenza surveillance data spanning from June 3, 2012, to May 27, 2018. Our objective is to unravel the potential interplay between virus migration and meteorological elements driving influenza A/H3N2 seasonality. This investigation aims to explain why influenza A/H3N2 typically exhibits summer/autumn epidemics in the subtropical/tropical zone, as opposed to the temperate zone [2,10,14,18,19]. Our findings will contribute scientific evidence to inform the development and refinement of influenza prevention and control strategies, not only in China but also in regions worldwide experiencing similar patterns of influenza seasonality across different temperature zones.

## 2 Materials and methods

### 2.1 Study data

#### 2.1.1 Influenza surveillance data

We gathered influenza surveillance data from China through the National Influenza Surveillance Network. Established in 1981, this network covers all municipalities in China as of 2009. It comprises 556 sentinel hospitals and 411 influenza network laboratories operated by the provincial and prefecture Centers for Disease Control and Prevention (CDC). However, the COVID-19 pandemic led to public health interventions such as social distancing, mask-wearing, and handwashing, which affected the natural pattern of influenza A/H3N2 activity [20]. To address this, we selected influenza surveillance data from June 3, 2012, to May 27, 2018, from mainland provinces in China. We excluded Tibetan data, which tested less than 10 % of weekly influenza specimens during the study period. Further processing of influenza surveillance data is shown in the Appendix A.

#### 2.1.2 Meteorological data

Meteorological data were sourced from China's Surface Climate Data (V3.0) daily value dataset, made available by the China Meteorological Administration (https://data.cma.cn/). This dataset encompasses essential weather variables, including daily temperature, precipitation, evaporation, relative humidity, wind direction and speed, sunshine hours, air pressure, and ground temperature at 0 cm, from 699 meteorological stations across China since January 1951. For our study, we focused on meteorological data spanning from June 3, 2012, to May 27, 2018, in Mainland China. More information is shown in the Appendix A.

#### 2.1.3 Gene sequence data

We acquired gene sequence data from the NCBI (National Center of Biotechnology Information, https://www.ncbi.nlm.nih.gov/genomes/FLU/Database/nph-select.cgi?go=database) and specifically chose H3–HA nucleotide sequences from human hosts in China during the period from June 3, 2012, to May 27, 2018. Any sequences with unclear province origins were omitted, resulting in a final dataset of 187 sequences.

### 2.2 Models for spatiotemporal distribution of influenza A/H3N2

#### 2.2.1 Fixed-effect regression model

We employed the individual fixed-effect regression model [21] to quantify the impact of the spatiotemporal distribution of each influenza subtype's positivity rate on the overall spatiotemporal distribution of influenza positivity rates in China. The model's formula is presented in the Appendix A. To assess the stationarity of panel data, including the positivity rates of each influenza subtype and the overall influenza positivity rate, we conducted the Im-Pesaran-Shin (IPS) panel unit root test, along with the Levin, Lin, and Chu (LLC) panel unit root test. All original data exhibited stationarity according to both the LLC and IPS tests.

To compare the contributions of individual influenza subtypes to the spatiotemporal distribution of influenza, we standardized each subtype's positivity rate through z-score transformation and used the model's slope as an indicator of their spatiotemporal impact. The model's results are presented in Table B1 in the Appendix B.

#### 2.2.2 Average threshold method

We employed the method developed by Azziz Baumgartner [22] to identify the influenza A/H3N2 epidemic period in both China's temperate and subtropical/tropical zones. The formula for calculating the influenza A/H3N2 epidemic threshold (${\mathrm{IPT}}_{d}$) is as follows:

(1) $${\mathrm{IPT}}_{d}=\frac{\sum_{i=1}^{s}{\mathrm{IPR}}_{d,i}}{s}$$

Where d and i represent the epidemiological regions and week, respectively. ${\mathrm{IPT}}_{d}\;$denotes the epidemic threshold for influenza A/H3N2 in region d, and ${\mathrm{IPR}}_{d,i}$ stands for the standardized positive rate of influenza A/H3N2 in week i of region d, as defined in (1), (2), (3) in appendix A. Additionally, s represents the number of weeks in each epidemic season, where s is 53 in leap years and 52 in non-leap years. Each epidemic season commences in the 23rd week and concludes in the 22nd week of the following year.

The onset of the epidemic period is identified as the first week with a positive rate higher than ${\mathrm{IPT}}_{n\;\mathrm{or}\;d}\;$for three consecutive weeks, while the end of the epidemic period is designated as the first week with a positive rate lower than ${\mathrm{IPT}}_{n\;\mathrm{or}\;d}$ for three consecutive weeks [22].

#### 2.2.3 Definition of the stable epidemic period

**Fig. 1:**
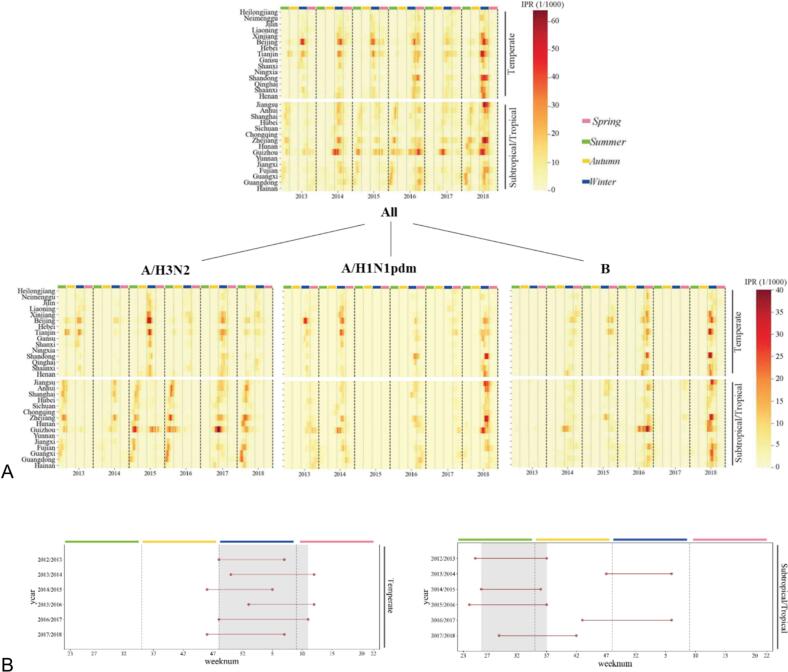
Epidemic patterns of influenza A/H3N2 in temperate and subtropical/tropical China were observed from June 2012 to June 2018. (A) Heatmaps displaying influenza and the positive rate of its types/subtypes by Chinese province. The time series of weekly influenza and its types/subtypes rate are arranged by increasing latitude from bottom to top. Black vertical lines mark the beginning and end of epidemic seasons, with each season starting at the 23rd week and ending at the 22nd week of the following year [2]. Green vertical lines indicate the four seasons of the year. (B) The onset and conclusion of each season's epidemic period and the stable epidemic period are shown. The red lines represent the epidemic period, and the dots at each end mark the starting and ending weeks of the epidemic period. The gray-shaded areas represent the stable epidemic periods observed during 2012–2018. (For interpretation of the references to colour in this figure legend, the reader is referred to the web version of this article.)

Referring to a previous study on stable influenza epidemic periods [23], we defined a stable epidemic week in temperate or subtropical/tropical zones as a week where more than half of the epidemic seasons in a given area [23] experienced the epidemic. If this condition persisted for three consecutive weeks, the area was considered to have entered a stable epidemic period (Fig. 1B).

### 2.3 Models for influence of virus migration

#### 2.3.1 Phylogenetic analysis

For phylogenetic analysis, including the construction of phylogenetic trees and the calculation of the timing of the Most Recent Common Ancestor (MRCA) [24], we utilized gene sequencing data and employed Bayesian [25] and Markov-chain Monte Carlo methods [26] through BEAST (Version 1.10.4). Due to the limited data available in this study, we opted for a strict clock to account for variations in the molecular evolutionary rate among lineages. We selected the HKY nucleotide substitution model and the Gamma site heterogeneity model [27]. As for the tree prior, we chose the Coalescent: Bayesian SkyGrid [28]. We sampled 1000 trees and relevant parameter estimates from each run, totaling 1000 generations.

#### 2.3.2 Bayesian SkyGrid reconstruction of past population dynamics

To reconstruct the relative genetic diversity of the influenza A/H3N2 virus in China's temperate and subtropical/tropical zones, we employed the Bayesian SkyGrid analysis available in Tracer v1.7.2 [28]. The SkyGrid model combines a Gaussian Markov random field smoothing scheme to infer past population dynamics from gene sequencing data.

### 2.4 Models for influence of meteorological elements

#### 2.4.1 Generalized additive model (GAM)

This study examined the influence of temperate and subtropical/tropical climate elements in China on the standardized positive rate of influenza A/H3N2 using the Generalized Additive Model (GAM) [29,30]. GAM is a versatile non-parametric statistical model that allows each independent variable to exhibit a distinct relationship with the dependent variable, avoiding the need for linear assumptions. In this analysis, the standardized positive rate of influenza A/H3N2 served as the dependent variable, while candidate independent variables included average temperature (AT, °C), precipitation (P, mm), average relative humidity (ARH), average wind speed (AWS, m/s), sunshine duration (SH, hours), and their interactions. To account for the typical one-week impact of meteorological factors on influenza-positive rates [31,32], the maximum lag time was set to one week. The specific settings of GAM model refer to Appendix A.

#### 2.4.2 Threshold analysis

Meteorological factors have a threshold effect on influenza epidemics [33]. To determine the threshold of meteorological elements for influenza A/H3N2, we conducted a generalized additive regression, with meteorological elements serving as independent variables and the standardized positive rate of influenza A/H3N2 in each temperature zone (IPR; see formula 3 in the appendix A) as the dependent variable. This ratio was divided by the average threshold during the epidemic period (IPT; see formula 1). According to the definition of the average threshold method, the influenza A/H3N2 epidemic commences when IPR > IPT. Therefore, the threshold for meteorological elements corresponds to the value when IPR/IPT equals 1.

## 3 Results

### 3.1 The spatiotemporal distribution of influenza A/H3N2 in China

Fig. 1A illustrates the distinct patterns of influenza in China's temperate and subtropical/tropical zones. In the temperate zone (provinces north of 33°N), influenza follows an annual winter epidemic pattern [34], while in the subtropical/tropical zone (provinces south of 33°N), there is a biannual epidemic pattern, occurring in summer/autumn and winter [34] as shown in Fig. 1B. This study also examined and compared the seasonality of different influenza types and subtypes. Influenza A/H1N1 and B primarily prevail during the winter across all temperature zones in China, aligning with the seasonality of influenza A/H3N2 in the temperate zone. However, in the subtropical/tropical zone, influenza A/H3N2 is predominantly active during summer or autumn. Subsequently, we utilized an individual fixed-effect regression model to the impact of the spatiotemporal distribution of each influenza subtype's positivity rate on the overall spatiotemporal distribution of influenza positivity rates in China (Table B1). The findings highlight that the contributions of the three subtypes are not significantly different. However, the activity of the A/H3N2 virus is the predominant cause of the influenza epidemic peaks during the summer and autumn in China's tropical and subtropical regions (showed in Fig. 1A). This peak has led to variations in influenza prevalence patterns across the country's different latitudinal zones. Therefore, the study aims to investigate the spatial distribution patterns and the driving mechanisms behind the seasonality of influenza A/H3N2.

Moving on to Fig. 1B, it provides a visualization of the patterns of influenza A/H3N2 seasonality, epidemic timing, and the duration of epidemics in the temperate and subtropical/tropical zones of China, calculated using the average threshold method. Evidently, distinct seasonality is observed between influenza A/H3N2 epidemics in these two temperature zones. In the temperate zone, influenza A/H3N2 epidemics typically commence in late autumn and early winter, persisting throughout the winter. According to the stable epidemic period results (Fig. 1B), the epidemic begins in the 48th week of the year (i.e., the fourth week of November) and ends in the 11th week of the following year (i.e., the second week of March). Conversely, in the subtropical/tropical zone, epidemics are primarily concentrated in the summer and autumn, occurring between the 26th and 37th weeks (i.e., the fourth week of June to the 24th week of September). Notably, there are no epidemics in any temperature zone in China during the 12th to 25th weeks (i.e., the third week of March to the third week of June) and the 38th to the 47th weeks (i.e., the fourth week of September to the third week of November).

### 3.2 The function of virus migration for seasonal epidemics of influenza A/H3N2 in China's temperature zones

**Fig. 2:**
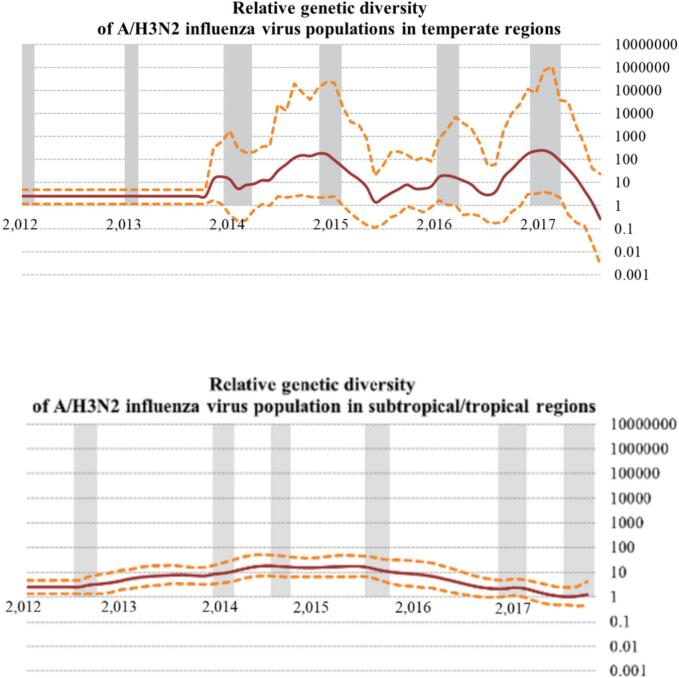
The Bayesian SkyGrid analysis reveals shifts in relative genetic diversity within China's temperate and subtropical/tropical zones from 2012 to 2018. On the x-axis, time spans from the youngest sampled sequence to the lower tree-root height, while the y-axis represents relative genetic diversity obtained from the SkyGrid coalescent analysis. A 95 % confidence interval is denoted by the yellow dashed line, and the gray-shaded background marks the stable epidemic period of influenza A/H3N2, which is calculated in formula 1 and shown in Fig. 1B. (For interpretation of the references to colour in this figure legend, the reader is referred to the web version of this article.)

To understand the population dynamics of influenza A/H3N2 in China's temperate and subtropical/tropical zones, this study utilized Bayesian “SkyGrid” reconstruction analysis to examine relative genetic diversity fluctuations between 2012 and 2018 (Fig. 2). The results indicated that the influenza A/H3N2 virus population in China's temperate zone exhibited significant seasonal fluctuations in relative genetic diversity, corresponding with seasonal prevalence. Each population bottleneck occurred at the end of each epidemic season, highlighting the annual need for new genetic diversity infusion.

**Fig. 3:**
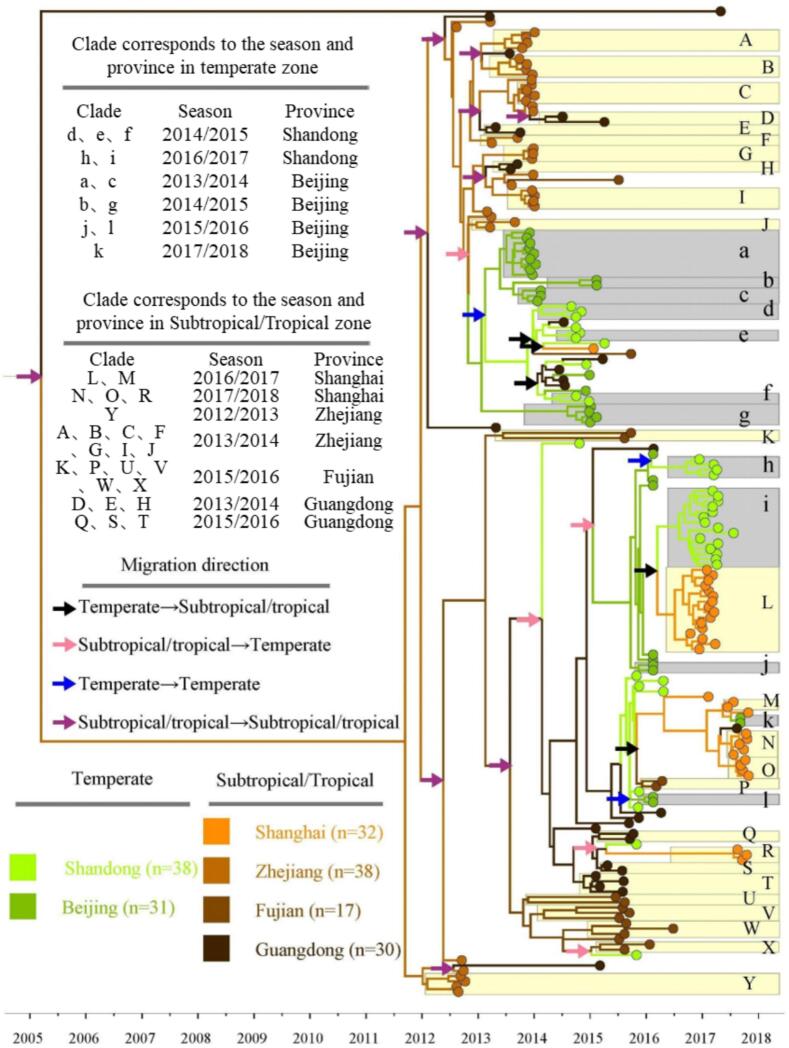
Phylogenetic tree of the influenza A/H3N2 viruses. This tree serves as a representation of Bayesian sampled trees. The gray rectangles specifically indicating the presence of these strains in temperate provinces, as denoted by lowercase alphabetical characters. In contrast, the pale yellow rectangles signify the subtropical and tropical provinces where the viral lineage is observed, distinguished by uppercase alphabetical characters. The variable “n” signifies the quantity of viral gene sequence samples in each province. The corresponding seasons and provinces for each clade are detailed in the table, along with information about the direction of migration between these clades. (For interpretation of the references to colour in this figure legend, the reader is referred to the web version of this article.)

As shown in Fig. 3, influenza A/H3N2 viruses isolated from various locations in China extensively mixed on the phylogenetic tree. However, the migration of the influenza A/H3N2 virus between China's temperate and subtropical/tropical zones was not direction-specific or path-dependent. Moreover, the subtropical/tropical zone did not serve as the source of the influenza A/H3N2 virus, contradicting the hypothesis of regular virus migration driving influenza A/H3N2 seasonality.

In addition, the only subtype to emerge from the 2014/2015 epidemic season in Beijing evolved from the a-subtype of the 2013/2014 epidemic season in the temperate zone. Similarly, in the subtropical/tropical zone, subtypes A, B, C, F, G, I, and J from the 2013/2014 epidemic season in Zhejiang evolved from subtype Y in the 2012/2013 epidemic season. And subtypes N and O from the 2017/2018 epidemic season in Shanghai evolved from subtype M in the 2016/2017 epidemic season. Except for these minor branches producing a few descendant viruses, all influenza A/H3N2 viruses circulating in provinces during each epidemic season were introduced from other provinces. This indicates that local evolution did not significantly contribute to generating genetic diversity [35]. Therefore, changes in genetic diversity and extensive geographic migration suggest that most H3N2 influenza viruses circulating in China's temperate zones during epidemic seasons primarily originate from the global gene pool via virus migration.

To further explore the origin of genetic diversity, this study also calculated the age of the most recent common ancestor (MRCA) of non-endemic virus subtypes collected from the same province and epidemic season, based on the phylogenetic tree (Appendix B, Table B2). The average MRCA range was 3.3 months (95 % confidence interval, 2.3–6.8 months). This relatively old MRCA value indicates that the ancestors of the influenza A/H3N2 virus population in China might have existed for several months or more before an epidemic season. Furthermore, this suggests that some influenza A/H3N2 virus variant populations did not immediately spread upon their introduction to China's temperate zones [35]. Instead, they only circulated when activated by driving factors.

### 3.3 The influence of meteorological elements to influenza A/H3N2 epidemic patterns between temperature zones in China

To investigate the impact of meteorological factors on seasonal influenza A/H3N2 prevalence, we established generalized additive models (GAM) for both China's temperate and subtropical/tropical zones. The results revealed that average weekly temperature significantly affected influenza A/H3N2 in the temperate zone, whereas the average temperature one week prior had a significant impact in the subtropical/tropical zone (refer to (2), (3), more details in appendix A).

For temperate zone (R-square = 0.43):

(2) $$\log\left(\mathrm{IPR}\right)=s\left(\mathrm{AT},4.26\right)-0.300\;$$

For subtropical/Tropical zone (R-square = 0.26):

(3) $$\log\left(\mathrm{IPR}\right)=s\left(\mathrm{AT}\left(t-1\right),5.87\right)+0.567$$

where IPR stands for the standardized positive rate of influenza A/H3N2, AT denotes the average temperature (°C), and t−1 refers to meteorological factors from one week prior. week represents the number of weeks in the research period, with the study covering weeks 1 to 313. $s\left(\mathrm{cov},\mathrm{edf}\right)$ represents the nonparametric nonlinear relationship between variable cov and IPR. edf indicates the estimated smooth degree of freedom.

**Fig. 4:**
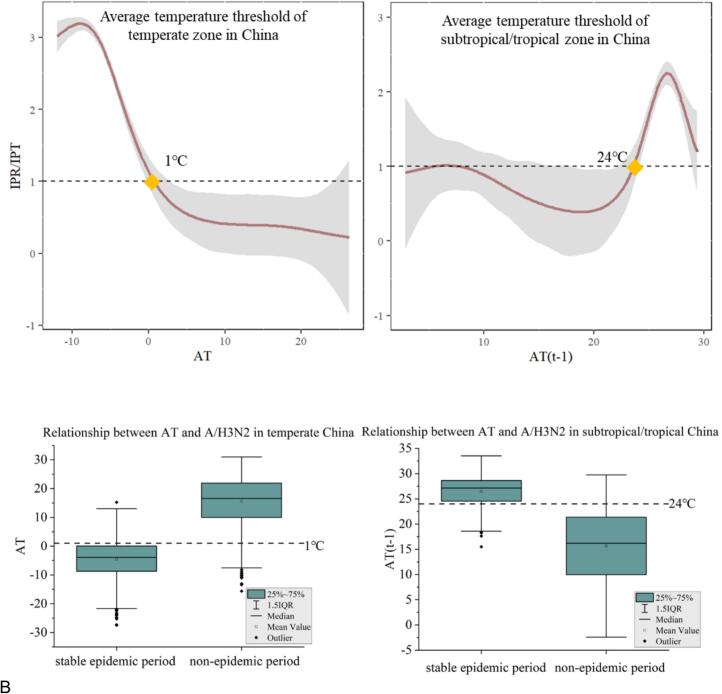
The threshold effect of weekly average temperature on influenza A/H3N2 incidence in both temperate and subtropical/tropical zones of China is depicted here. In (A), the x-axis represents the current week's average temperature or that of the preceding week, while the y-axis represents IPR/IPT. The gray shading represents the 95 % confidence interval, and the yellow diamond indicates the average temperature corresponding to the average threshold. (B) shows the temperature distribution during stable epidemic and non-epidemic periods in temperate and subtropical/tropical provinces of China. Stable epidemic periods encompass the winter epidemic phase (from the 48th to the 11th week of the following year) in temperate zones and the summer epidemic phase (from the 26th to the 37th week) in subtropical/tropical zones, with the remaining time considered as non-epidemic periods. (For interpretation of the references to colour in this figure legend, the reader is referred to the web version of this article.)

Based on threshold effect analysis, in the temperate zone, when the average weekly temperature dropped below 1 °C (95 % confidence interval: 0–3 °C), the standardized positive rate of influenza A/H3N2 exceeded the average threshold, signaling the onset of influenza A/H3N2 transmission. Conversely, in the subtropical/tropical zone, when the average weekly temperature one week prior rose above 24 °C (95 % confidence interval: 22–24 °C), the standardized positive rate of influenza A/H3N2 surpassed the average threshold, indicating the beginning of influenza A/H3N2 transmission (Fig. 4A).

By analyzing the average temperature distribution during stable and non-epidemic periods of influenza A/H3N2 in each Chinese province, we found that the mean temperature threshold could effectively determine the start and end dates of the epidemic in each province (Fig. 4B). In temperate provinces, the influenza A/H3N2 epidemic period (from the 48th week to the 11th week of the following year) only occurred when the average temperature fell below 1 °C. Similarly, subtropical/tropical provinces experienced an influenza A/H3N2 epidemic (between the 26th and 37th weeks) when the average temperature one week prior was above 24 °C. Furthermore, the average temperature during non-epidemic periods in all temperature zones in China never exceeded the lower limit of 1 °C or the upper limit of 24 °C.

In summary, meteorological elements activate the influenza A/H3N2 virus following migration, contributing to the spatial distinction in A/H3N2 epidemic patterns between temperature zones in China.

## 4 Discussion

In this study, we investigated the spatial distribution of influenza A/H3N2 seasonality in different temperature zones in China and examined the underlying mechanisms. We observed significant spatial variations in influenza A/H3N2 seasonality between China's temperate and subtropical/tropical zones. This seasonality appeared to be influenced by a combination of factors, including virus migration and meteorological elements. Furthermore, the migration of the A/H3N2 virus and its response to meteorological variations exhibited distinct temporal delays. The latency period for the virus's reaction to meteorological factors was considerably shorter compared to the duration of its migration.

In summary, it seemed that A/H3N2 influenza virus populations did not primarily evolve within specific regions to drive seasonal epidemics. Instead, there was a continuous exchange of virus variants across regions, even among locations separated by distinct temperature zones. This cross-region virus migration introduced new viral populations, essentially sowing the “seeds” for subsequent seasonal epidemics. Meteorological changes, both in terms of space and time, appeared to activate latent influenza A/H3N2 viruses, leading to seasonal epidemics in different temperature zones in China [33,[36], [37], [38], [39], [40]].

When a new influenza virus lineage emerges in a host, the host's immune system responds, leading to increased resistance to the virus [36]. The new outbreaking lineage benefits from a larger infection volume and higher infection efficiency but also faces heightened competition from older lineages due to the host's enhanced immune response. As herd immunity grows, emerging lineages will gain a competitive edge and replace older ones [35,37,38].

Regarding A/H3N2 virus migration, our findings align with global patterns [11,41,42]. Typically, antigenic variants of A/H3N2 viruses from previous epidemics rapidly decline, while new variants from different regions sustain local chains of infection. New antigenic variants often emerge following epidemics caused by existing variants [43].The ability of the influenza A/H3N2 virus to spread is influenced greatly by its aggressiveness and its capacity for rapid replication and host invasion. Viruses must transport their genetic material across a lipid envelope through specific structural proteins to infect host cells [44]. The physical properties of viral envelope lipids depend on the infection's temperature. The influenza virus enters host cells through endocytosis, which can be influenced by the cellular environment. Viral stability below 10 °C promotes lipid ordering, critical for airborne transmission [43]. Therefore, the rapid spread of latent influenza A/H3N2 viruses depends on the ambient environment.

Influenza A/H3N2 seasonality likely correlates with meteorological elements, a key environmental factor that influences virus migration and survival, considering host immunity and behavior [17]. Temperature variation can explain the spatial heterogeneity of epidemics, a phenomenon observed in many infectious diseases [[44], [45], [46]]. Thresholds, such as 1 °C and 24 °C, appear to be important in explaining the seasonal epidemics of influenza A/H3N2 in China's temperate and subtropical/tropical zones.

To infect host cells, the virus must protect its genetic material with an envelope and deliver it into host cells [47]. When budding from the host cell's plasma membrane, the virus selects an ordered lipid structural domain as the envelope, and the orderliness of the lipid structure relates to the ambient temperature during host infection. When the temperature drops below 10 °C, the virus's lipids gradually begin to organize, promoting the formation of the envelope and accelerating the spread of the virus [43]. Another study has clearly revealed that as the temperature rose from 25 °C to 42 °C, the proportion of ordered lipids in intact viruses would gradually decrease, and the proportion of ordered lipids was almost nonexistent at temperatures above 40 °C [48]. In China's temperate zone, where temperatures drop below freezing in winter, the orderly arrangement of the virus's lipid envelope facilitates airborne transmission, leading to prolonged seasonal epidemics. When spring arrives and temperatures rise, the virus becomes less stable and loses its transmission efficiency [33], marking the end of the influenza A/H3N2 epidemic.

In contrast, China's subtropical/tropical zone experiences hot and humid conditions with limited air circulation. High temperatures, sometimes exceeding 40 °C, prevent lipid ordering in the virus, resulting in low genetic diversity and minimal seasonal variation. Here, host factors appear to play a more significant role in the seasonal epidemics of influenza A/H3N2. Moriyama and Ichinohe found that exposing mice to a high-temperature environment would impair the migration of dendritic cells that secrete pro-inflammatory cytokines to lymph nodes, aggravate virus-specific CD8 T-cells, and stimulate antibody responses after influenza virus infection due to increased lung tissue autophagy [49]. Therefore, high-temperature environments can impact the host's immune response and adaptability to the influenza A/H3N2 virus, explaining why epidemics in this zone typically peak in summer and autumn.

In addition, the evolution of influenza viruses, particularly the H3N2 subtype, is significantly influenced by temperature changes. Research indicates that the temperature sensitivity of these viruses undergoes regular, cyclical variability during natural drift, which is crucial for assessing their evolutionary and epidemic potential [50]. Therefore, global warming may impact the transmission and burden of influenza. While climate change may reduce the intensity of influenza epidemics by increasing specific temperature, it could also prolong the duration and year-round evolution potential at different latitudes.

Similar patterns of influenza seasonality and the role of meteorological elements have been observed in other countries and regions with distinct temperature zones. A study conducted across 11 sites worldwide assessed the impact of meteorological variations on influenza subtypes. It revealed that cold temperatures primarily drove influenza A activity in temperate regions, while subtropical and tropical areas exhibited a U-shaped relationship between temperature and influenza A epidemics [40]. In temperate countries like Canada, where the average temperature threshold was around 7 °C [40], and South Korea, with a threshold of 0 or 5 °C [51], the low-temperature threshold closely aligned with these findings. Another global study identified a critical high-temperature threshold for influenza outbreaks at 24 °C [33]. Subtropical and tropical countries such as Thailand, Guatemala, El Salvador, and Panama showed a positive correlation between temperature and influenza incidence when the average temperature exceeded 25–29 °C [38].

The patterns of influenza A/H3N2 epidemics are expected to change in response to global climate change. According to the projections from the Intergovernmental Panel on Climate Change's sixth assessment report (https://www.ipcc.ch/report/ar6/wg1/), global warming is anticipated to reach or exceed 1.5 °C in the next 20 years. Consequently, in the future, the onset of influenza A/H3N2 epidemics in China's temperate zone is likely to be delayed, whereas in the subtropical/tropical zone, they may occur earlier.

However, it's important to acknowledge the limitations of this study. The surveillance period was relatively short, which may have hindered the detection of long, stable periods of influenza A/H3N2 epidemics. In the subtropical/tropical zone of China, summer/autumn influenza A/H3N2 epidemics have occurred for several years, suggesting that various factors, such as topography, population movement, and the activity of other influenza virus subtypes [36,52], may have played roles not fully considered. Additionally, despite robust influenza surveillance in many Chinese provinces, insufficient gene sequencing and associated metadata limited the comprehensive and detailed analysis of the spatiotemporal evolutionary dynamics of influenza A/H3N2 viruses. It should be noted that the findings of this study are derived from an ecological analysis revealing a mathematical correlation. Further multidisciplinary research is warranted to validate the synergistic effects of virus migration and meteorological factors, as well as to explore the mechanisms of high temperature's impact on human health.

## 5 Conclusion

Enhanced surveillance is crucial for monitoring influenza A/H3N2 and other strains across varying temperature zones. Animals play various roles in A/H3N2 virus migration, acting as potential carriers, amplification hosts, intermediaries, and vectors. To prevent zoonotic spillover events and ensuing human outbreaks, comprehensive monitoring of influenza viruses in both human and animal populations is also essential.

Climate change has the potential to alter the timing of influenza outbreaks, making it crucial for policymakers and health agencies to adapt. This adaptation may involve adjusting vaccination schedules and improving public health communication. In regions where temperature significantly influences influenza trends, targeted interventions are vital during peak seasons. These measures could include promoting hygiene, mask usage, and social distancing in high-risk periods.

Continuous research is essential to understand the complex factors affecting influenza seasonality from a “One Health” perspective, which involves recognizing the interconnectedness of human health, animal health, and the environment. Given the global significance of influenza and its relationship with climate change, international cooperation is indispensable for effectively monitoring and managing outbreaks. Through information sharing and the adoption of best practices, we can collectively reduce the global impact of influenza.

## CRediT authorship contribution statement

**Yilan Liao:** Writing – review & editing, Methodology, Conceptualization. **Tong Zhao:** Writing – original draft, Software, Formal analysis. **Wei Du:** Writing – review & editing. **Dayan Wang:** Methodology, Conceptualization. **Zhibin Peng:** Methodology, Conceptualization. **Shan Xue:** Investigation, Formal analysis. **Jianxing Yu:** Methodology, Conceptualization. **An Zhang:** Writing – original draft. **Hongyan Ren:** Writing – original draft. **Zhoupeng Ren:** Writing – original draft. **Geoge Fu Gao:** Writing – review & editing. **Jinfeng Wang:** Writing – review & editing. **Nils Christian Stenseth:** Writing – review & editing.

## Declaration of competing interest

The authors declare that they have no known competing financial interests or personal relationships that could have appeared to influence the work reported in this paper.

## Data Availability

The influenza surveillance datasets analyzed during the current study are available from China's CDC. However, restrictions apply to the availability of these data, which were used under license for the current study. Correspondingly, they are not publicly available. Other data analyzed during this study are included in this article.

## Appendix A Supplementary data

Supplementary material 1 Image 1

Supplementary material 2 Image 2
