# Supplementary material for: How virus migration and meteorological elements shape the seasonality of influenza a/H3N2: A case study in China
Source: One Health. 2025 Apr 14;20:101037. doi: 10.1016/j.onehlt.2025.101037 (PMC12020868; doi:10.1016/j.onehlt.2025.101037)
Supplement: Supplementary file 1 — Supplementary material 1 [file mmc1.docx]

**Appendix A. Supplementary material**

1. **Influenza surveillance data**

To estimate the true incidence, we calculated the influenza A/H3N2 positive rate (${IPR}_{n,i}$) using the ILI%*LAB method [1]. We then aggregated the Chinese temperature zone scale for the positive influenza A/H3N2 rate (${IPR}_{d,i}$) by summing each province's weekly positive rate in the zone, with the province's population as the weight factor. The formula of ${IPR}_{n,i}$ and ${IPR}_{d,i}$ is as follows:

${LAB}_{n,i}=P_{n,i}/N_{n,i}$ (1)

${IPR}_{n,i}={ILI}_{n,i}*{LAB}_{n,i}$ (2)

${IPR}_{d,i}=\sum_{n\epsilon D} {IPR}_{n,i}*\frac{{pop}_{n}}{\sum_{n\epsilon D} {pop}_{n}}$ (3)

where $n$ represents provinces, $i$ represents weeks, and $N_{n,i}$ and $P_{n,i}$ denote the number of influenza specimens tested and the number of influenza-positive isolates of province $n$ in the $i^{th}$ week, respectively. Moreover, ${ILI}_{n,i}$ is the ratio of patients from sentinel clinics and hospitals in province $n$ during the $i^{th}$ week, who had a body temperature exceeding 38.0°C and exhibited symptoms of a cough or sore throat. In addition, $D$ represents the set of provinces within the same zone $d$, and ${pop}_{n}$ corresponds to the population estimate based on China’s sixth population census for province $n$.

1. **Individual Fixed-effect regression model**

To appraise the individual contributions of influenza subtypes and types to the spatiotemporal dynamics of influenza prevalence, we employed an individual fixed effects model to quantify these influences. This model, a variant of the fixed effects framework, is distinguished by its assumption that the individual effect remains invariant over time. The schematic representation of the general model is delineated as follows.

$y_{it}=\beta X_{it}+\alpha_{i}+u_{it}$ (4)

Where $y$ represents the dependent variable, overall spatiotemporal distribution of influenza positivity rates, respectively. $X$ represents the independent variable, which is the spatiotemporal distribution of different influenza subtypes. $i and t$ represent the $i$th individual subtypes and time $t$, respectively. $\alpha$ represents the individual fixed effects, in this case the impact of the spatiotemporal distribution. $u$ represents the error term.

1. **Meteorological data**

To derive weekly meteorological data for each station, we employed SQL Server 2012 to aggregate daily information on temperature, precipitation, relative humidity, wind speed, and sunshine hours on a weekly basis. We calculated the weekly averages for temperature, relative humidity, wind speed, precipitation, and sunshine hours. Subsequently, we consolidated the weekly meteorological data for each province, with all meteorological elements summarized as averages. Tibet's data was excluded to maintain consistency with influenza surveillance data.

1. **Generalized additive model (GAM)**

A Generalized Additive Model (GAM) is an adaptable regression framework that permits non-linear interactions between individual predictors and the response variable, accommodating a variety of functional forms. Its general structure is outlined below.

$Y=\beta_{0}+\beta_{1}X_{1}+f\left( X_{2} \right)+\ldots+f(X_{p})$ (5)

where $Y$ represents the dependent variable, respectively. $p$ denotes the total number of independent variables. The independent variables are represented as $X_{1},X_{2},\cdots,X_{p}$. Specifically, $X_{1}$ exhibits a linear association with $Y$, whereas the remaining variables $X_{2},\cdots,X_{p}$ each have a nonlinear relationship with $Y$.

To consider potential time trends, we included the calendar week as a candidate independent variable. We constructed the GAM using the mgcv package (Version 1.8–35) within the R statistical programming environment. We defined the f(x) function of the GAM as a nonlinear, non-parametric smoothing function constructed using cubic spline functions, with the degrees of freedom (k) adaptively determined through Generalized Least Angle Regression (GAMLR) combined with Bayesian Information Criterion (BIC). We assumed a Gaussian distribution for the dependent variable, using the log as the link function. To address potential collinearity among independent variables, variables with a correlation exceeding 0.7 were excluded. Lasso screening was conducted using the Gemnet package (Version 4.1–7) to select the minimum penalty coefficient (lambda.1se) within one standard error, evaluated using mean squared error as the criterion through 10-fold cross-validation.

Finally, we assessed all possible candidate models to choose the most appropriate model with a cocurveability (worse) less than 0.7 [2], significant variable coefficients, and the best goodness of fit. The resulting GAM formulas are as follows:

For temperate zone (R-square = 0.43):

$log\left( IPR \right)=s\left( AT,4.26 \right)-0.300$ (6)

For subtropical/Tropical zone (R-square=0.26):

$log\left( IPR \right)=s\left( AT\left( t-1 \right),5.87 \right)+0.567$ (7)

where $IPR$ stands for the standardized positive rate of influenza A/H3N2, $AT$ denotes the average temperature (°C), and $t-1$ refers to meteorological factors from one week prior. $week$ represents the number of weeks in the research period, with the study covering weeks 1 to 313. $s(cov,edf)$ represents the nonparametric nonlinear relationship between variable $cov$ and $IPR$. $edf$ indicates the estimated smooth degree of freedom.

**Reference**

1. Wong JY, Peng W, Hiroshi N, Edward G, Lau EHY, Lin Y, et al. Infection Fatality Risk of the Pandemic A(H1N1)2009 Virus in Hong Kong. Am J Epidemiol. 2013;177(8):834-40.

2. Booth GD, Michael J. Niccolucci, and Ervin G. Identifying proxy sets in multiple linear regression : an aid to better coefficient interpretation. Ogden, UT: US Dept of Agriculture, Forest Service, Intermountain Research Station. 1994. P12.
