# Supplementary material for: How virus migration and meteorological elements shape the seasonality of influenza a/H3N2: A case study in China
Source: One Health. 2025 Apr 14;20:101037. doi: 10.1016/j.onehlt.2025.101037 (PMC12020868; doi:10.1016/j.onehlt.2025.101037)
Supplement: Supplementary file 2 — Supplementary material 2 [file mmc2.docx]

**Appendix B. Supplementary figures and tables**
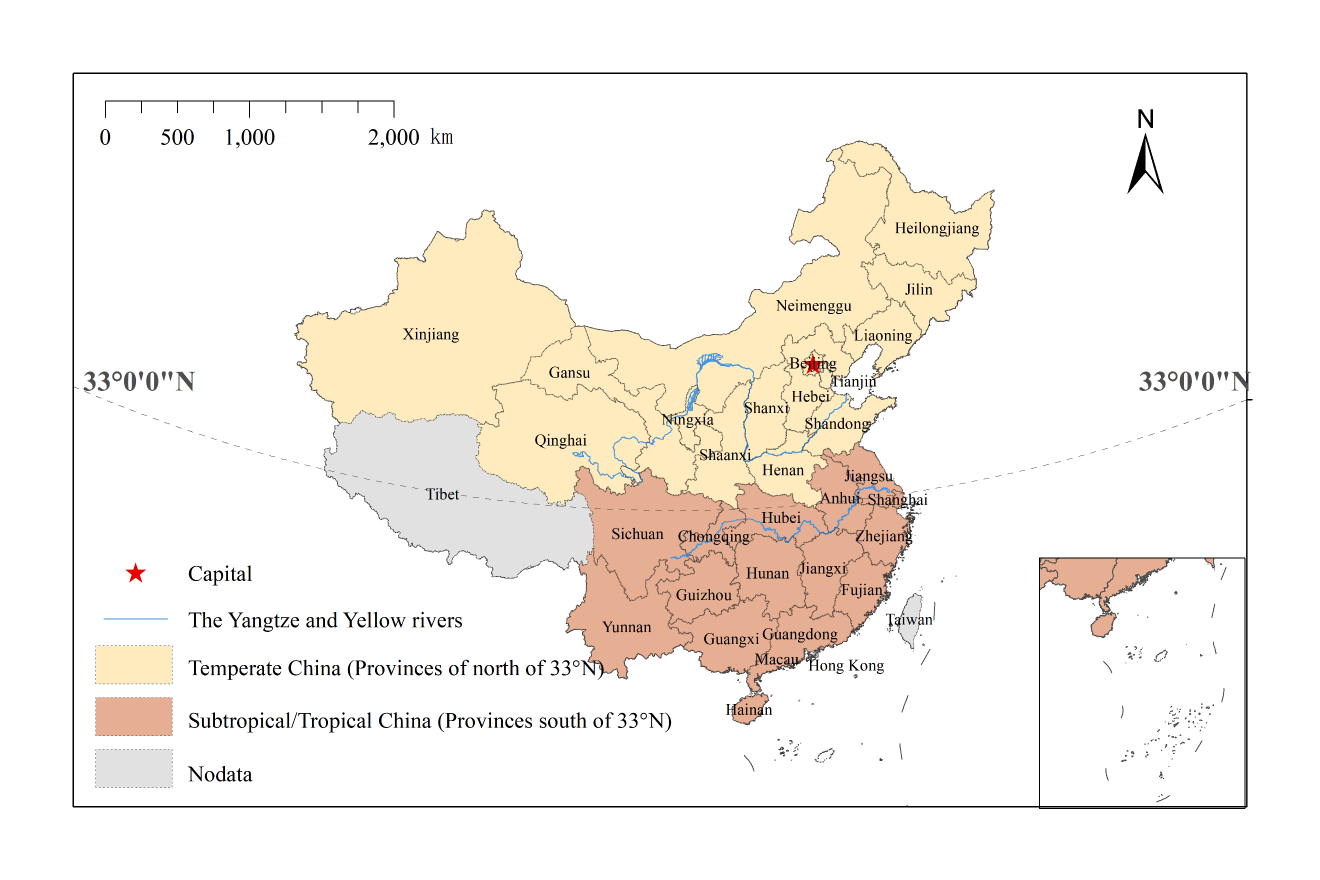


**Fig. B1.** Study’s area and zoning. The yellow area corresponds to temperate China, covering provinces located north of 33°N. The pink area represents subtropical/tropical China, encompassing provinces situated south of 33°N. Any areas not included in these zones are represented in white and were not studied.

**Table B1.** Contribution of each influenza subtype virus to the spatiotemporal distribution of overall influenza

|  | Subtype/Type | Contribution  (Normalized regression coefficient) |
| --- | --- | --- |
| Influenza | A/H1N1 | 0.45^*^ |
|  | A/H3N2 | 0.55^*^ |
|  | B | 0.50^*^ |

* represents p<0.001

**Table B2.** Months of MRCA by Clades

| Clade | Months of MRCA (95% CI) |
| --- | --- |
| L | 6.7 (3.8–20.5) |
| N | 4.9 (3.7–5.9) |
| O | 5.4 (4.1–6.5) |
| R | 5.3 (4.2–6.3) |
| T | 3.2 (3.1–3.2) |
| V | 1.7 (1.4–3.4) |
| Y | 0.8 (0.8–0.9) |
| a | 2.7 (0.9–13.6) |
| c | 0.6 (0.5–1.2) |
| d | 1.1 (1.1–1.2) |
| g | 0.9 (0.6–2.1) |
| h | 2.9 (2.5–3.1) |
| i | 6.6 (3.0–21.0) |
